# Estimating population access to insecticide-treated nets from administrative data: correction factor is needed

**DOI:** 10.1186/1475-2875-12-259

**Published:** 2013-07-26

**Authors:** Albert Kilian, Hannah Koenker, Lucy Paintain

**Affiliations:** 1 Tropical Health LLP, Montagut, Girona, Spain; 2 Johns Hopkins Bloomberg School of Public Health Center for Communication Programs, Baltimore, MD, USA; 3 Department of Disease Control, London School of Hygiene & Tropical Medicine London, London, UK

## Abstract

**Background:**

Population access to insecticide-treated nets (ITN) is usually determined from survey data. However, for planning purposes it is necessary to estimate this indicator between surveys. Two different approaches are currently recommended for such estimates from administrative data, multiplying the number of ITN delivered either by 2.0 or 1.8 before dividing by the population. However, the validity of such estimates has not previously been investigated.

**Methods:**

Thirty-five datasets from household surveys in sub-Saharan Africa were selected from ten different countries. The number of ITN and *de-facto* population from the samples was used as proxy administrative data and estimates of population access to ITN were calculated using the recommended formulae. Administrative estimates were compared to the access indicator from the survey data. Regression analysis was used to further define the relationship between administrative and survey population access. Mean number of ITN users was determined for each data set separately for households with and without enough ITN.

**Results:**

Analysis of users per ITN showed that the assumption of two users per net is valid overall (median 2.00) but that it was consistently lower in households with at least one ITN for every two people (median 1.66). Using the formula number of ITN times 2.0 divided by the population to estimate population access to ITN from administrative data generally overestimated the survey access indicator. This was particularly the case at higher coverage levels, resulting in a 30 percentage-point overestimate at survey access above 80%. Using 1.8 as the multiplier for the number of ITN from administrative data improved the results but still showed a 19 percentage-point overestimate at access coverage above 80%. Regression analysis found that a factor of 1.64 provides the best prediction of the access indicator with slight underestimation at low access levels but good fit at levels above 55%.

**Conclusions:**

A factor of 1.6 rather than 2.0 or 1.8 as the mean number of users per ITN provides a more accurate estimation of population access to ITN from administrative data accounting for discordant ITN-person pairs and a reduced number of ITN users when sufficient ITN are available.

## Background

With the renewed focus on the possibility of malaria elimination, vector control through universal coverage with insecticide-treated nets (ITN) has become one of the core elements of the Global Malaria Action Plan [1] and measuring its progress is crucial. The World Malaria Report 2012 [2] uses the indicator of “access to long-lasting insecticidal nets (LLIN) within the household” as one of the key metrics in line with recent recommendations by the Roll Back Malaria Monitoring and Evaluation Reference Group (MERG) [3]. Access is defined as the number of potential net users in the household, assuming that each net protects two people, and that nets cannot protect more people than currently live in a household [4]. This ITN access indicator not only assesses the level of population coverage but also provides a way to directly compare the percentage of actual net users the previous night against the percentage of people who could have used a net, allowing a more accurate measure of the ‘net use gap’ by eliminating those who are not using nets because they do not have access to them.

Data on ITN coverage and use is generally obtained from large household surveys such as demographic and health surveys (DHS), multiple indicator cluster surveys (MICS) or malaria indicator surveys (MIS). However, these surveys usually only take place every two to three years and often malaria programme managers need to monitor coverage in a more continuous fashion or at lower administrative levels not covered by the surveys. In addition, estimates of population coverage with ITN are needed for planning of continuous distribution strategies or to plan the timing of a “top-up” campaign. One way of obtaining such interim estimates is to use administrative data, namely the number of ITN distributed and the target population from census or other sources.

A number of Roll Back Malaria partnership documents provide suggestions on how to calculate such administrative coverage: the Alliance for Malaria Prevention toolkit [5] recommends estimating ITN access following a mass distribution campaign in the absence of a household survey by multiplying the total number of ITN delivered during a campaign by a factor of 2.0 and then dividing by the total population. This is based on the general assumption that on average two people share a net. The Harmonization Working Group Country Briefing for Global Fund Round 11 [6] recommends multiplying the number of ITN distributed or assumed still to be present at time X by the factor 1.8 to estimate administrative population coverage. The factor of 1.8 as opposed to 2.0 is based on the recommendation by Kilian and others [7] to use this factor when planning macro-quantification for LLIN needs for universal coverage. This adjustment increases the number of nets needed for full universal coverage by accounting for odd-numbered households where two individuals cannot share a net because they reside in different households, which is referred to as a ‘discordant net-person pair’ in this paper.

Neither of these approaches has previously been validated to show how accurate they are compared to survey estimates of population access to ITN within the household. However, there is theoretical ground to suspect that neither of them would be accurate. The 1.8 factor is based on the proportion of odd-numbered households and only refers to 100% coverage; in contrast, the access estimation refers to the population and here the proportion of discordant net-person pairs may differ and vary depending on level of coverage. One way of validating these formulae is to use the number of ITN and population from representative surveys as a proxy for administrative data and compare estimates of administrative access using different formulae to the access metric obtained from the original, individual level survey data. Because real life administrative data have additional sources of bias such as inaccurate census data and incorrect estimation of the true number of ITN in the population, use of such proxy administrative data is a conservative approach and it can be expected that discrepancies between survey results and administrative estimates will differ even more in real life situations. However, as the level of error in the administrative data is usually not known, the approximation with survey data seems the best way to explore the systematic relationship between these measures.

This paper explores the ways in which existing recommendations on use of administrative data to estimate population ITN access relate to results from survey data and how they could be improved. The strength and weaknesses of such recommendations are discussed from both scientific and pragmatic, managerial perspectives.

## Methods

### Survey datasets

A total of 35 survey datasets from sub-Saharan Africa were purposively selected for analysis. To be included, surveys had to be standard, cluster-sampling household surveys, representative nationally or for a well-defined subnational administrative unit (region, state, province, etc.), use of the standard questionnaire modules for malaria as recommended by MERG [4] or equivalent, and the original data had to be accessible in order to obtain all necessary indicators for analysis. Attempts were made to include the whole spectrum of access coverage from very low to very high values as well as to cover different areas of Africa (Table 1). This implied that all recent surveys with likely high access results were included and those left out were those from the earlier years when ITN coverage was low. All national survey datasets were downloaded with permission from the Measure DHS website except for the Mozambique MIS (obtained from Malaria Consortium). Additional subnational survey data were provided by Malaria Consortium except for three Ghana surveys (Brong Ahafo, Central and Western regions) which were implemented by the London School of Tropical Medicine and Hygiene (LSTMH) and Dodowa Health Research Centre on behalf of UNICEF.

**Table 1:** Datasets used in the analysis

**Country**	**Location (region, state or district)**	**Survey type**	**Year**	**Number of ITN in sample**	***De-facto *population in sample**	**ITN per 100 people**	**% of population with access to ITN**
Ghana	Northern	Post-campaign	2010	1,612	6,332	25.5	47.0
Ghana	Central	Post-campaign	2012	1,090	2,903	37.5	62.1
Ghana	Western	Post-campaign	2012	1,255	3,046	41.2	66.3
Ghana	Eastern	Post-campaign	2012	2,278	5,052	45.1	74.4
Ghana	Brong Ahafo	Post-campaign	2012	2,055	3,526	58.3	86.4
Liberia	National	MIS	2009	3,552	21,876	16.2	25.4
Liberia	National	MIS	2011	3,293	18,632	17.7	30.8
Malawi	National	MIS	2012	2,932	14,091	20.8	37.2
Madagascar	National	MIS	2011	12,454	39,337	31.7	57.3
Mozambique	National	MIS	2007	1,229	27,360	4.5	8.6
Mozambique	Sub-national*	Project baseline	2010	2,851	11,957	23.8	40.3
Nigeria	Kano	Post-campaign	2009	1,108	4,602	24.1	44.0
Nigeria	Anambra	Post-campaign	2009	1,540	4,462	34.5	50.1
Nigeria	National	MIS	2010	4,909	30,088	16.3	28.7
Nigeria	Niger	Post-campaign	2010	1,259	6,188	20.3	34.4
Nigeria	Ogun	Post-campaign	2010	706	4,030	17.5	36.8
Nigeria	Sokoto	Post-campaign	2010	1,219	4,424	27.6	49.1
Nigeria	Katsina	Post-campaign	2010	1,498	4,562	32.8	56.1
Nigeria	Nasarawa	Post-campaign	2011	1,116	5,008	22.3	41.5
Nigeria	Cross River	Post-campaign	2011	1,254	5,441	23.0	45.9
Senegal	National	MIS	2006	2,776	28,918	9.6	17.5
Senegal	National	MIS	2008	17,997	88,257	20.4	34.9
Senegal	Sub-national **	Post-campaign	2011	6,605	15,290	43.2	75.2
South Sudan	Lainya	Post-campaign	2011	709	3,324	21.3	37.9
Tanzania	National	DHS	2004	3,828	46,416	8.2	15.6
Tanzania	National	AIS	2007	8,005	42,517	18.8	25.4
Tanzania	National	DHS	2010	13,045	47,357	27.5	46.6
Tanzania	National	AIS	2011	23,092	50,192	46.0	74.8
Uganda	National	DHS	2006	2,220	43,396	5.1	9.1
Uganda	Sub-national***	Project baseline	2009	1,369	20,037	6.8	11.6
Uganda	National	MIS	2009	3,758	20,918	18.0	31.6
Uganda	Kamuli	Post-campaign	2010	5,648	12,048	46.9	65.3
Uganda	Sub-national***	Post-campaign	2010	1,501	2,724	55.1	80.9
Uganda	National	DHS	2011	11,742	43,508	27.0	44.7
Uganda	Sub-national***	Project midterm	2011	2,824	8,727	32.4	58.2

*MIS* malaria indicator survey, *DHS* demographic and health survey, *AIS* AIDS indicator survey, *ICCM* integrated community-based case management.

* provinces Inhambane, Nampula, Cabo Delgado.

** regions Kolda, Sedhiou, Tambacounda, Kedougou, Kaolack, Kaffrine.

*** districts Buliisa, Hoima, Kyankwanzi, Kiboga, Kyenjojo, Masindi, Kibaale.

### Data definition and analysis

All data management and analysis was done using STATA version 11.2 (STATA Corporation, College Station, Texas, USA) or Excel 2010 (Microsoft Corporation, Seattle, Washington, USA). All analyses accounted for survey design including sampling weights where applicable using the “svy” command family in STATA.

As a proxy for administrative data two metrics were used: i) the total number of ITN owned by households in the survey defined as either an LLIN identified by the brand label or a net treated with insecticide within the previous 12 months; ii) the *de-facto* population in the sample, i e, all people present in the household the night before the survey irrespective of whether they were usual household members or visitors. These data were entered into an Excel database and the estimated access from administrative data was calculated for each survey by multiplying the number of ITN in the sample by either 2.0 or 1.8 and then dividing by the *de-facto* population. In addition, a variable was created for the ratio between ITN and population per survey expressed as ITN per 100 population.

The survey indicator of access to ITN within the household was calculated from the datasets of individual household members as recommended by MERG [3,4]. First, an intermediate variable of “potential ITN users” was created by multiplying the number of ITN in each household by a factor 2.0. In order to adjust for households with more than one net for every two people the potential ITN users were set equal to the *de-facto* population in that household if the potential users exceeded the number of people in the household. Second, the access indicator was calculated by dividing the potential ITN users by the number of *de-facto* members for each household and determining the overall sample mean of that fraction.

Mean number of users per used ITN was determined by first creating a dataset with nets found in the household as the unit of observation where it did not yet exist. For each net user listed by line number from the household register a dichotomous variable was created indicating 1 if any person was listed as user and 0 if not and the sum of these user variables for each net was calculated to obtain the total number of users per net. The mean users for those nets identified as ITN and used by anyone the previous night were obtained using sampling weights where appropriate and taking account of the design effect using the “svy” command family in STATA. The same analysis was done for the subgroups of population living in households that did or did not have at least one ITN for every two people.

The relationship between the estimated administrative access to ITN with the factor 2.0 or 1.8 and the survey access indicator was explored using linear and fractional polynomial regression analysis (“fracpoly” command in Stata). Similarly, linear regression analysis using ITN/100 people as the predictor of the survey access result was applied to identify the optimal correction factor for the administrative data.

## Results

Details of the 35 datasets used are given in Table 1. The year of implementation ranged from 2004 to 2012 with the majority (83%) from 2009 or later. Ten countries were included from west (Senegal, Liberia, Ghana, Nigeria), east (South Sudan, Uganda, Tanzania) and southern Africa (Malawi, Mozambique, Madagascar) with the largest number of surveys being from Nigeria (nine), Uganda (seven) and Ghana (five). Seventeen surveys (49%) were post-campaign evaluation surveys, 15 surveys (43%) were national-level DHS, MIS or AIDS indicator surveys (AIS), and the remaining three were project baseline or midterm evaluation surveys. Five surveys (14%) were subnational, 13 (37%) covered a single state, province or region and two (6%) a single district (Kamuli District in Uganda, Lainya County in South Sudan).

The median *de-facto* population per survey was 12,048 with range from 2,724 (post-campaign survey Western Uganda 2010) to 88,257 (Senegal MIS 2008); the median number of ITN per survey was 2,278 with range from 706 (post-campaign survey Ogun State, Nigeria 2010) to 22,663 (AIS Tanzania 2011); and the ratio between the two expressed as ITN/100 people ranged from 4.5 to 58.3 with a median of 23.4.

Median proportion of the *de-facto* population with access to an ITN within the household from survey data was 44.0% with a range from 8.6% (MIS Mozambique 2007) to 86.4% (Brong Ahafo Region, Ghana 2012) with a reasonable distribution between these points (Table 1).

### Number of users per ITN used

**Figure 1:**
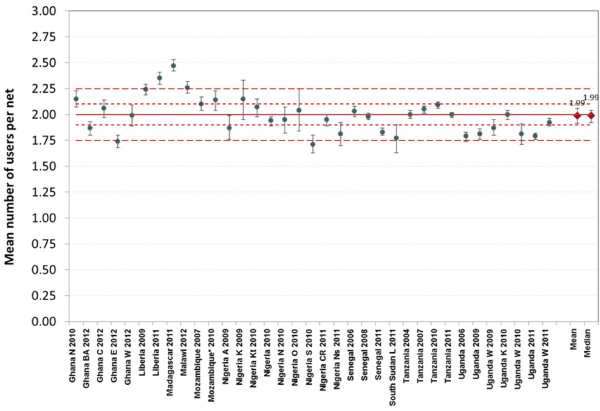
**Mean number of users per ITN used the previous night.** Error-bars represent 95% confidence intervals; long-dashed lines range 1.75 to 2.25; short-dashed lines range 1.90 to 2.10.

Given that the primary outcome variable of “access to ITN within the household” is based on the assumption that an ITN is on average shared by two people, the first step in the analysis was to explore to what extent this assumption holds. The mean number of users per ITN for all used ITN varied between 1.71 and 2.47 (Figure 1 and Additional file 1). The mean across the 35 datasets was 1.99 (95% confidence interval (CI): 1.93, 2.05), and the respective median was 1.99 (inter-quartile range (IQR) 1.83, 2.09). Using ±0.10 ITN users as acceptable variation, i e, 1.90 to 2.10 users/used ITN, 16 datasets (46%) were within these limits rising to 25 (71%) if the criterion was that the 95% CI of the survey results included the acceptable range of variation (1.90 to 2.10 users per ITN used). When the acceptable variation was set to ±0.25 users/ITN, i e, 1.75 to 2.25, 30 datasets (86%) were within these limits and 33 (94%) if inclusion within the 95% CI was considered. As shown in Figure 1, the results within the same country seemed to be quite similar except for Ghana and Nigeria where the data came from different parts of the country and a considerable variation was seen.

**Figure 2:**
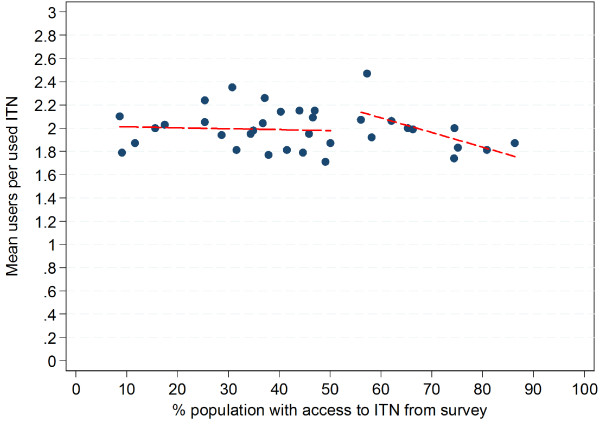
**Mean number of users per ITN used the previous night plotted against population access to ITN from survey.** Long-dashed line represents linear regression for values below 55% access; short dashed line regression above 55% access.

**Figure 3:**
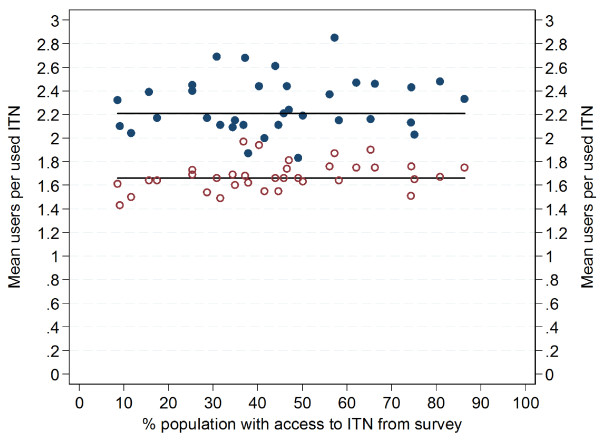
**Mean number of users per ITN used the previous night disaggregated by household supply with ITN.** Full circles households with less than one ITN for every two people; open circles households with one ITN for every two people or more. Horizontal lines represent the respective median for each group.

Plotting the mean number of ITN users per net against the population access to ITN from the survey data did not overall show any trend (Figure 2) and regression analysis confirmed this with a co-efficient of −0.001 (p=0.3, R-squared 0.002). However, when only surveys with a population access to ITN of 55% or more were considered a moderate linear decline was evident with a co-efficient of −0.13 (p=0.03, R-squared 0.37) suggesting a declining number of ITN users per net at higher levels of access. In order to further explore this aspect, survey data on mean users per used ITN was calculated separately for those living in households with at least one ITN for every two people, i.e, enough ITN to cover all household members and those with an insufficient number of ITN. This revealed a systematically and significantly lower number of ITN users when enough ITN were available (Figure 3 and Additional file 1) with a mean of 1.68 (95% CI 1.63, 1.71) and a median of 1.66 (IQR 1.61, 1.75) compared to mean 2.27 ITN users (95% CI 2.20, 2.36) and median 2.21 (IQR 2.11, 2.44) if the household did not have enough ITN. The two-sample t-test for comparison of the means was highly significant (t=−16.5, p<0.00001). The proportion of ITN that were shared by two people was similar in both groups (41.2% and 42.3%, mean across all 35 datasets) but among those from households with insufficient ITN only 20.8% of ITN were used by just one person and 38.0% by three or more while this was reversed once enough ITN were available in the household with 46.1% of ITN used by only one person and 11.7% by three or more.

### Relationship between survey and administrative access data applying the factor of 2.0 as mean ITN users

**Figure 4:**
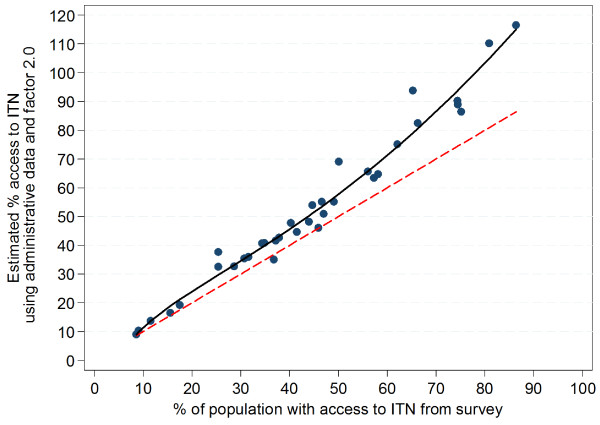
**Estimation of ITN access indicator using administrative data and the factor 2.0.** Red dashed line=equity; solid black line=polynomial function describing relationship of administrative estimate to survey access indicator.

The relationship between the access variable from the survey data and the estimation from administrative data using the factor 2.0 is shown in Figure 4 (exact figures in Additional file 2). In only one data set was the estimate obtained from administrative data higher than the survey estimate. Six data points (17%) were within ±2 percentage-points of the survey estimate and all of these were at survey access results of 50% or less. For all other surveys the estimate from administrative data was higher than the survey result and this overestimation increased with increasing access coverage reaching 11–16 percentage-points for survey access 70-80% and 29–30 percentage-points for survey access of 80-90%.

Fractional polynomial linear regression of the administrative results (A_2.0_) against the survey results (S) showed the best fit for this relationship to be a model with two terms of the powers 0 (logarithmic) and 2 (square):

A_2.0_= 50.7 + 13.8 (ln(S/10)-1.49) + 1.0 ((S/10)^2^-19.6

The fit for this model was excellent with an R-squared of 0.97 and the gain in deviance (variance ratio F) was significant compared to a linear model (p=0.03) or one with only one power term (p=0.01).

### Relationship between survey and administrative access data applying the factor of 1.8 as mean ITN users

**Figure 5:**
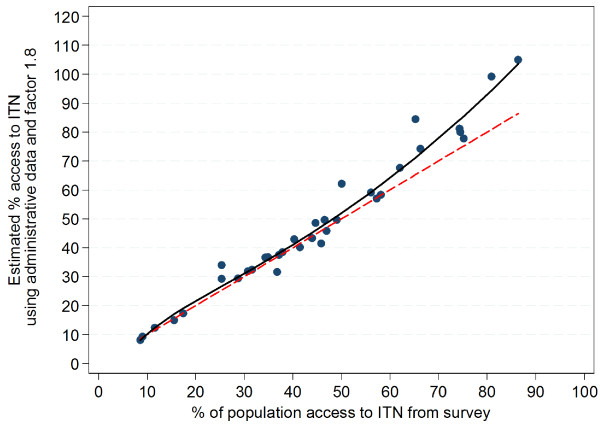
**Estimation of ITN access indicator using administrative data and the factor 1.8.** Red dashed line=equity; solid black line=polynomial function describing relationship of administrative estimate to survey access indicator.

Estimating access to ITN from administrative data using the factor 1.8 as the multiplier shows results much closer to the survey results with most values below 50% access coverage from surveys directly or close to the equity line (Figure 5 and Additional file 2). However, above 50% survey coverage the estimates from administrative data again increasingly overestimated the survey result, albeit to a lesser degree with values 5–8 percentage-points higher for survey access 70-80% and 18–19 percentage-points too high for survey access 80-90%.

Fractional polynomial regression gives the same degree (2), power terms (0, 2) and best fit (R-squared 0.97) previously obtained for estimation with the factor 2.0 only with different co-efficients:

A_1.8_= 45.6 + 12.5 (ln(S/10)-1.49) + 0.90 ((S/10)^2^-19.6

### Determining an appropriate correction factor for administrative data

**Figure 6:**
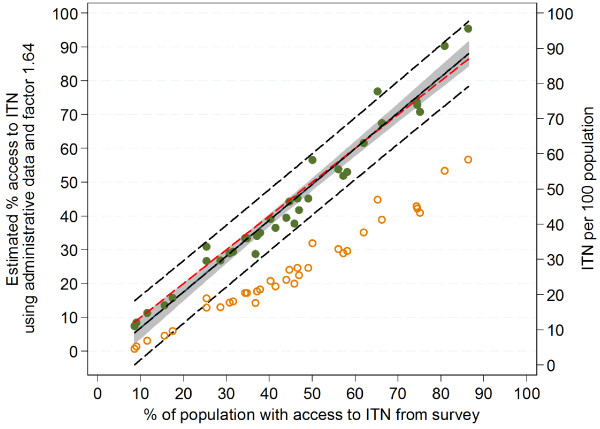
**Estimation of ITN access indicator using administrative data and the factor 1.64.** Red dashed line=equity for survey access; solid black line=linear function describing relationship of estimate to access indicator; gray area=95% confidence interval for regression line; dashed black lines=95% confidence interval of predictions; green closed circles=predicted access indicator values, orange open circles= administrative data expressed as ITN/100 people.

Plotting the original administrative data expressed as ITN/100 people against the survey results of the survey access variable (Figure 6) shows that in contrast to estimations using the factors 2.0 or 1.8, the ratio between ITN and *de-facto* population has a reasonably linear relationship with the survey access indicator results. This suggests that the overestimation of the access indicator from administrative data is based on an increasing mismatch of the factors 2.0 and 1.8 at higher levels of access coverage. It also suggests that a better multiplier which would give a good result across all levels of access coverage could be defined by a linear regression model using ITN/100 people as a predictor of “% population with access to ITN”, forcing the regression line through zero (no constant option) in order to avoid estimating access when there are no nets in the population. This regression gives a co-efficient of 1.64 (95% CI: 1.58, 1.69) and an excellent model fit (R-squared 0.99, p<0.00005). As shown in Figure 6 the regression line is slightly above equity for survey access coverage above 55% and below the equity line for lower values with the result that at access coverage below 35%, the estimation from administrative data using the factor 1.64 underestimates the survey coverage by 1–2 percentage-points (Additional file 2) and 1–8 percentage-points for survey coverage 35% to 55% but avoids an overestimation at coverage 70 to 80% and significantly reduces it at coverage 80 to 90%; with the adjustment factor of 1.64, the overestimation at 80 to 90% is 8–9 percentage-points compared to 29–30 percentage-points for estimation using the factor 2.0 and 18–19 percentage-points using factor 1.8.

## Discussion

Using the total number of ITN and population from representative household survey data sets as a proxy for administrative data this study explored the accuracy of two different, currently recommended approaches to estimate the “access to ITN within the household” indicator from available administrative data between surveys by comparing such estimates with the actual survey results for 35 datasets from sub-Saharan Africa.

Results indicate that using a factor of 2.0 as a multiplier for the number of ITN before dividing by the population generally overestimates access, particularly at levels of access coverage above 50% resulting in an overestimation of up to 30 percentage-points and results clearly above 100% at survey access levels above 80% (Figure 4 and Additional file 2).

Interestingly, data from the surveys suggests that the principle assumption of an average of two users per ITN underlying the formula is actually quite accurate at ITN level as the median of the “mean number of users per used ITN” indicator across the 35 surveys was 1.99 and 71% of estimates were consistent with a mean user range of 1.90 to 2.10, i e, their 95% confidence intervals fell within this range. One of the reasons for the increasing overestimation of access when looking at the population level is the occurrence of discordant net-person pairs where a potential second net user is located in a different household making sharing of the net physically impossible. This leads to a functional mean of ITN users per used ITN at population level below 2.0. Although the proportion of potential discordant net-person pairs can be expected to be constant in a given population as it is driven by the proportion of households with an odd number of family members, the impact of this factor on the estimated access of the population to ITN from administrative data will be relatively small if net ownership and access is low but will increase with increasing population coverage as seen in the data (Figure 2). The issue of odd-numbered households has previously been identified as a problem for the macro-quantification of ITN for campaigns resulting in a revised factor of 1.78 rather than 2.0 based on analysis of 18 survey datasets [7] which resulted in the current formula of “population/1.8” for the number of ITN needed to achieve universal coverage recommended by WHO [8].

The second reason for increasing disparity between the “access to ITN” indicator from survey data and administrative data using the factor 2.0 at higher coverage levels is the lower number of users per ITN once a household has sufficient ITN for all members. In these situations almost half (46.1%) of the ITN were used by only one person and only 11.7% by three or more bringing the median number of users per used ITN down to 1.66. As the proportion of households with sufficient ITN for all members increases at high coverage levels reaching between 40 and 60% (data not shown) this contributes to the observed moderate decline of the mean ITN users at access levels above 55% (Figure 2). A higher proportion of single ITN users once sufficient ITN are available within the household is very plausible considering that older/oldest members of the household and children over age five are more likely to have their own sleeping spaces and also have been shown to be least likely to be sleeping under a net when there are not enough nets [9]. As these family members, who generally use single bed sleeping arrangements, are added to the users, the overall average of users per net decreases.

Using the factor 1.8 to estimate the proportion of people with access to ITN from administrative data [6] leads to a much better fit compared to a factor of 2.0 with good results for access coverage below 50%; however, there is still increasing overestimation at higher access coverage of up to 19 percentage-points (Figure 3). The factor 1.8 was based on a simulated allocation of “enough” nets to all households and an estimation of the resulting ratio between population and ITN needed when 100% of households had “one ITN for every two people”. In the case of the access indicator estimation, however, the reference is the population, not households and it appears that the proportion of people living in odd-numbered households is slightly higher than the proportion of households with an odd number of members leading – in conjunction with the lower mean user value at full coverage – to the observed overestimate even when the factor 1.8 was used.

The regression analysis of the ratio of ITN and population found in the surveys against the survey-derived access indicator provides the figure of 1.64 as the optimal compromise to predict population ITN access from administrative data avoiding significant overestimation at high coverage rates, although at the price of slight underestimation at levels below 60% access coverage (Figure 4). Given that most countries in sub-Saharan Africa now approach access coverage of 40 to 60% [2] a slight underestimation at lower coverage levels seems justified if it leads to more accuracy at higher coverage levels which are the most critical for planning purposes for continuous distributions or follow-up distributions campaigns.

In this study the number of ITN found in the survey sample and the *de-facto* survey populations have been used as a proxy for administrative data. This is not realistic, as usually the ITN numbers would be obtained from records of previous distributions discounted by estimated losses due to wear and tear [6] and a population estimate from previous census data or other sources such as immunization records. Both of these data sources are prone to considerable inaccuracies, for example, if net durability differs from the assumed rates or if significant changes such as in- or out-migration or reduction in population growth rates have occurred since the last census. In general it can be assumed that real life administrative data tend to further overestimate the true ITN access of the population, but in each individual case a prediction is difficult to make as the magnitude and direction of the bias is not known. In contrast, the figures used in this study are much more accurate reflecting the exact number of ITNs and people on the day of the survey. This means that the relationship between administrative data and survey results in this analysis is more exact than if true administrative data were used and the variations seen in the results should be considered conservative compared to a real-life situation. One way to address this issue would be a modelling exercise that allows a sensitivity analysis of the impact of various degrees of over- and underestimation of the real life administrative data on the access indicator estimation. Such modelling, however is beyond the scope of this study and can be suggested for future research.

There is little doubt that there is a great need to estimate progress towards universal coverage with ITN from administrative data between surveys or following distribution campaigns either by project and programme managers or in the context of projections of global progress [2]. Using a formula that significantly overestimates the true, survey-derived access indicator could have negative effects by suggesting a coverage level that actually does not exist, leading to discrepancies between expected and actual survey results, thereby discrediting the efforts of malaria control. On the other hand, any change in current practices must be simple enough to be broadly applied and it is therefore suggested that a formula for the interim estimation of access of population to ITN is used as follows:

% population with ITN access= number of ITN * (1.6/target population)*100

This revised formula will still be sufficiently accurate considering the previously discussed potential variations in true administrative data and should be considered by the relevant structures of WHO as a recommendation to malaria programmes and the international community at large.

This study has several limitations. First, the selection of surveys to be included was purposive even though all recent surveys were included and it cannot be excluded that the variations in results would be somewhat larger had all available data sets from the past years be included. It is, however, very unlikely that this would have a significant impact on the regression co-efficient and hence would not have altered the resulting recommendation. Second, only five datasets at access coverage above 70% were available and only two above 80%, which proved to be the critical area of overestimation. While it clearly would be desirable to have more data points at high access coverage levels, such data currently do not exist and a re-analysis of the situation may be needed at a later point in time. But again, it is not very likely that this would dramatically alter the current estimate of 1.6 being the best compromise as the multiplier.

## Conclusions

To estimate administrative population coverage of ITN after or between household surveys based on number of available or distributed ITN and the target population, a factor of 1.6 mean users per ITN provides a more accurate prediction of population access to ITN accounting for the presence of discordant ITN-person pairs at household level and a reduced number of users per ITN when sufficient ITN are available for all household members.

## Competing interests

The authors declare that they have no competing interests.

## Authors’ contributions

AK designed the study; AK, HK and LP participated in the data analysis and interpretation. All authors contributed to the final version of the text and have read and approved the manuscript.

## Supplementary Material

Additional file 1: Table 2 Results for mean number of ITN users. Click here for file

Additional file 2: Table 3 Results from estimations for each data set sorted by the survey access variable. Click here for file
