# Supplementary material for: Estimating population access to insecticide-treated nets from administrative data: correction factor is needed
Source: Malar J. 2013 Jul 26;12:259. doi: 10.1186/1475-2875-12-259 (PMC3726288; doi:10.1186/1475-2875-12-259)
Supplement: Additional file 1: Table 2 — Results for mean number of ITN users. [file 1475-2875-12-259-S1.pdf]

Additional File 1:

Table 2: Results for mean number of ITN users

Country	Location (Region, State or District)	Year	Mean users	95% Confidence Interval for mean users	Mean users if not 1 ITN per 2 people	Mean users if 1 ITN per 2 people
Ghana	Northern	2010	2.15	2.07 to 2.23	2.24	1.81
Ghana	Central	2012	2.06	1.96 to 2.14	2.33	1.75
Ghana	Western	2012	1.99	1.89 to 2.09	2.47	1.75
Ghana	Eastern	2012	1.74	1.68 to 1.80	2.13	1.51
Ghana	Brong Ahafo	2012	1.87	1.80 to 1.93	2.49	1.75
Liberia	National	2009	2.24	2.19 to 2.29	2.40	1.73
Liberia	National	2011	2.35	2.29 to 2.41	2.69	1.66
Malawi	National	2012	2.26	2.21 to 2.32	2.85	1.87
Madagascar	National	2011	2.47	2.42 to 2.53	2.68	1.68
Mozambique	National	2007	2.10	2.04 to 2.23	2.32	1.61
Mozambique	Regional*	2010	2.14	2.04 to 2.23	2.44	1.94
Nigeria	Kano	2009	1.87	1.76 to 1.99	2.19	1.63
Nigeria	Anambra	2009	2.15	1.63 to 1.80	2.61	1.66
Nigeria	National	2010	1.71	1.89 to 1.98	2.37	1.76
Nigeria	Niger	2010	1.94	1.82 to 2.07	2.17	1.54
Nigeria	Ogun	2010	1.95	1.84 to 2.25	2.09	1.69
Nigeria	Sokoto	2010	2.04	1.84 to 2.25	2.11	1.97
Nigeria	Katsina	2010	2.07	1.98 to 2.15	1.83	1.66
Nigeria	Nasarawa	2011	1.81	1.70 to 1.92	2.21	1.66
Nigeria	Cross River	2011	1.95	1.89 to 2.00	2.00	1.55
Senegal	National	2006	2.03	1.98 to 2.08	2.17	1.64
Senegal	National	2008	1.98	1.95 to 2.01	2.15	1.60
Senegal	National	2011	1.83	1.80 to 1.87	2.03	1.65
South Sudan	Lainya	2011	1.77	1.63 to 1.90	1.87	1.62
Tanzania	National	2004	2.00	1.96 to 2.04	2.39	1.64
Tanzania	National	2007	2.05	2.01 to 2.08	2.45	1.69
Tanzania	National	2010	2.09	2.06 to 2.12	2.44	1.74
Tanzania	National	2011	2.00	1.97 to 2.02	2.43	1.76
Uganda	National	2006	1.79	1.74 to 1.83	2.10	1.43
Uganda	West	2009	1.81	1.76 to 1.86	2.11	1.49
Uganda	National	2009	1.87	1.80 to 1.95	2.04	1.50
Uganda	Kamuli	2010	1.81	1.71 to 1.91	2.16	1.90
Uganda	West	2010	2.00	1.95 to 2.04	2.48	1.67
Uganda	National	2011	1.92	1.89 to 1.96	2.11	1.55
Uganda	West	2011	1.79	1.76 to 1.82	2.15	1.64

* Provinces Inhambane, Nampula, Cabo Delgado
