# Supplementary material for: Estimating population access to insecticide-treated nets from administrative data: correction factor is needed
Source: Malar J. 2013 Jul 26;12:259. doi: 10.1186/1475-2875-12-259 (PMC3726288; doi:10.1186/1475-2875-12-259)
Supplement: Additional file 2: Table 3 — Results from estimations for each data set sorted by the survey access variable. [file 1475-2875-12-259-S2.pdf]

Additional File 2:

Table 3: Results from estimations for each data set sorted by the survey access variable

Country	Location (Region, State or District)	Year	% of population with access to ITN	Estimate using ITN*2.0/ pop	Estimate using ITN*1.8/ pop	Estimate using regression without constant+
Mozambique	National	2007	8.6	9.0	8.1	7.3
Uganda	National	2006	9.1	10.2	9.2	8.3
Uganda	Sub-national***	2009	11.6	13.7	12.3	11.1
Tanzania	National	2004	15.6	16.5	14.8	13.5
Senegal	National	2006	17.5	19.2	17.3	15.7
Liberia	National	2009	25.4	32.5	29.2	26.5
Tanzania	National	2007	25.4	37.7	33.9	30.7
Nigeria	National	2010	28.7	32.6	29.4	26.6
Liberia	National	2011	30.8	35.3	31.8	28.8
Uganda	National	2009	31.6	35.9	32.3	29.3
Nigeria	Niger	2010	34.4	40.7	36.6	33.2
Senegal	National	2008	34.9	40.8	36.7	33.3
Nigeria	Ogun	2010	36.8	35.0	31.5	28.6
Malawi	National	2012	37.2	41.6	37.5	33.9
South Sudan	Lainya	2011	37.9	42.7	38.4	34.8
Mozambique	Sub-national*	2010	40.3	47.7	42.9	38.9
Nigeria	Nasarawa	2011	41.5	44.6	40.1	36.4
Nigeria	Kano	2009	44.0	48.2	43.3	39.3
Uganda	National	2011	44.7	54.0	48.6	44.0
Nigeria	Cross River	2011	45.9	46.1	41.5	37.6
Tanzania	National	2010	46.6	55.1	49.6	44.9
Ghana	Northern	2010	47.0	50.9	45.8	41.5
Nigeria	Sokoto	2010	49.1	55.1	49.6	44.9
Nigeria	Anambra	2009	50.1	69.0	62.1	56.3
Nigeria	Katsina	2010	56.1	65.7	59.1	53.6
Madagascar	National	2011	57.3	63.3	57.0	51.8
Uganda	Sub-national***	2011	58.2	64.7	58.2	52.8
Ghana	Central	2012	62.1	75.1	67.6	61.3
Uganda	Kamuli	2010	65.3	93.8	84.4	76.5
Ghana	Western	2012	66.3	82.4	74.2	67.2
Ghana	Eastern	2012	74.4	90.2	81.2	73.6
Tanzania	National	2011	74.8	92.0	82.8	72.5
Senegal	Sub-national **	2011	75.2	86.4	77.8	70.5
Uganda	Sub-national***	2010	80.9	110.2	99.2	89.9
Ghana	Brong Ahafo	2012	86.4	116.6	104.9	95.1

* provinces Inhambane, Nampula, Cabo Delgado

** regions Kolda, Sedhiou, Tambacounda, Kedougou, Kaolack, Kaffrine

*** districts Buliisa, Hoima, Kyankwanzi, Kiboga, Kyenjojo, Masindi, Kibaale

+ access=1.64*(ITN/100 people)
